# ASSESSMENT OF RISK FACTORS FOR SURGERY TREATMENT OF CROHN’S DISEASE: A HOSPITAL COHORT

**DOI:** 10.1590/0102-672020230002e1730

**Published:** 2023-05-12

**Authors:** João Batista Monteiro TAJRA, José Ulisses CALEGARO, Silvana Marques e SILVA, Dannilo Brito SILVEIRA, Liliana Moscoso RIBEIRO, Stefane Mariano CRISPIM, Matheus EMERICK, João Victor Ribeiro TAJRA

**Affiliations:** 1 Hospital de Base do Distrito Federal, Coloproctology – Brasília (DF), Brasil;; 2 Hospital de Base do Distrito Federal, Research Management – Brasília (DF), Brasil;; 3 Hospital de Base do Distrito Federal, Epidemiological Monitoring – Brasília (DF), Brasil.; 4 University Center Unieuro, Medicine – Brasília (DF), Brasil.

**Keywords:** Crohn disease, Inflammatory bowel disease, Colorectal surgery, Doença de Crohn, Doenças inflamatórias intestinais, Cirurgia colorretal

## Abstract

**BACKGROUND:**

New therapies have revolutionized the treatment of Crohn’s disease (CD), but in some countries, the surgery rate has not changed, the frequency of emergency surgery is underestimated, and surgical risk is poorly studied.

**AIMS:**

The aim of this study was to identify risk factors and clinical indications for primary surgery in CD patients at the tertiary hospital.

**METHODS:**

This was a retrospective cohort of a prospectively collected database of 107 patients with CD from 2015 to 2021. The main outcomes were the incidence of surgery treatment, types of procedures performed, surgical recurrence, surgery free time, and risk factors for surgery.

**RESULTS:**

Surgical intervention was performed in 54.2% of the patients, and most of the procedures were emergency surgeries (68.9%). The elective procedures (31.1%) were performed over 11 years after diagnosis. The main indications for surgery were ileal stricture (34.5%) and anorectal fistulas (20.7%). The most frequent procedure was enterectomy (24.1%). Recurrence surgery was most common in emergency procedures (OR 2.1; 95%CI 1.6–6.6). Montreal phenotype L1 stricture behavior (RR 1.3; 95%CI 1.0–1.8, p=0.04) and perianal disease (RR 1.43; 95%CI 1.2–1.7) increased the risk of emergency surgeries. The multiple linear regression showed age at diagnosis as a risk factor for surgery (p=0.004). The study of surgery free time showed no difference in the Kaplan-Meier curve for Montreal classification (p=0.73).

**CONCLUSIONS:**

The risk factors for operative intervention were strictures in ileal and jejunal diseases, age at diagnosis, perianal disease, and emergency indications.

## RESUMO

**RACIONAL:**

Novas terapias revolucionaram o tratamento da Doença de Crohn, mas em alguns países a taxa de cirurgias não se modificou, a frequência de cirurgias de emergência é subestimada e o risco cirúrgico é pouco estudado.

**OBJETIVOS:**

Analisar fatores de risco e as indicações para cirurgia primária em pacientes com doença de Crohn, em Hospital terciário.

**MÉTODOS:**

Análise de banco de dados coletado prospectivamente, de 107 pacientes com doença de Crohn de 2015 a 2021. Os principais desfechos foram a incidência de tratamento cirúrgico, tipos de procedimentos realizados, recidiva cirúrgica, tempo livre de cirurgia e fatores de risco.

**RESULTADOS:**

A intervenção cirúrgica foi realizada em 54.2% dos pacientes, sendo a maioria dos procedimentos cirúrgicos de urgência (68.9%). Os procedimentos eletivos (31.1%) foram realizados mais de 11 anos após o diagnóstico. As principais indicações cirúrgicas foram estenose ileal (34,5%) e fístula anorretal (20,7%), sendo o procedimento mais frequente a enterectomia (24,1%). As cirurgias para recidiva foram mais comuns em procedimentos de urgência (OR 2,1; IC95% 1,6–6,6). O fenótipo de comportamento estenosante Montreal L1 (RR = 1,3; IC 95%: 1,0-1,8, p=0,04) e a doença perianal (RR 1,43; IC95% 1,2–1,7) aumentaram o risco de cirurgias de emergência. A regressão linear múltipla mostrou a idade ao diagnóstico como fator de risco para cirurgia (p=0,004). O estudo do tempo livre de cirurgia não mostrou diferença na curva de Kaplan Meier para a classificação Montreal (p=0,73).

**CONCLUSÕES:**

Os principais fatores de risco para intervenções cirúrgicas foram estenoses em doenças ileais e jejunais, idade ao diagnóstico, doença perianal e indicações de urgência.

## INTRODUCTION

Crohn’s disease (CD) is a chronic inflammatory disease of the gastrointestinal tract characterized by periods of clinical remission alternating with periods of recurrence. Persistent subclinical inflammation during clinical remission is thought to lead to complications and progressive bowel damage^9^. Management of CD is complicated by the transmural nature and patchiness of the disease, which make it difficult to observe the disease activity^18^. Complications such as strictures, fistulas, and dysplasia result from ongoing inflammation, and the risk factors to prevent the development of flares or surgical intervention are not clear^6^.

The incidence of inflammatory bowel disease is increasing in newly industrialized countries of South America and Asia^12,24^. The estimated annual incidence of CD in Brazil is between 0.84 and 3.5/10^5^ person-years with a prevalence of 2.4–14.1 cases/100,000 inhabitants^19,23^.

The effect of the available therapeutic approaches on the eventual need for surgery to treat CD is controversial. Some studies have reported a clear decrease in the resection rate in the past few decades^17,21^. However, a European population-based study found no difference in the rate of surgery in the first 5 years after diagnosis today compared with 20 years ago^7^, and a study in the United States reported that the rate of emergency surgery has remained unchanged over the years^11^. The onset of CD is generally insidious, and it occasionally presents as a fulminant disease with overlapping manifestations that require emergency treatment. Experienced surgical teams are needed for making disease-management decisions and preventing complications^15^. Currently, approximately 40% of CD patients are estimated to undergo surgery within 10 years after diagnosis. Long-term follow-up of an Australian cohort reported increases in emergency surgery and the rate of surgical recurrence in the decades following diagnosis^29^.

A recent association study showed that the location of CD can account for some of the risks in specific populations. In that study, the time from diagnosis to progression that required intervention surgical was significantly shorter in ileal (L1) compared with ileocolonic (L3) or colonic (L2) disease^8^. The ileal disease is associated with CD phenotypes involving stricture and obstruction that result in a more complicated disease and an increased need for surgery^14^. In recent decades, developments in drug therapy have significantly postponed the need for surgery^22^. Nevertheless, most of the patients with CD still require one or more surgical interventions during their disease.

The aim of this study was to identify factors that increase the risk of initial elective or emergency surgery in CD patients in the midwest region of Brazil.

## METHODS

A retrospective cohort study included 107 CD patients with regular follow-ups in the coloproctology unit of a tertiary hospital from 2015 to 2021. Patients diagnosed with CD with at least 12 months of follow-up and complete medical data were eligible for the study. Patients aged less than 14 years, without a confirmed diagnosis or having ulcerative colitis, were excluded.

The patients were stratified by the interval between diagnosis and surgery, beginning with diagnosis during surgery and continuing with 5-year intervals to 20 years or more. Patient data were recovered from an electronic database of medical records, including colonoscopies, surgical procedures, and pathology reports using the keywords such as Crohn’s disease, Crohn, and CD. The analyzed variables were patient clinical features, illness time, emergency indication, Montreal classification, perianal disease, and epidemiological profile. The primary outcomes were the incidence of major abdominal and perineal surgeries, free time to surgery, types of procedures performed, surgical recurrence, and main phenotypes linked to surgery.

Surgical procedures were classified as major abdominal or perianal. Major abdominal surgeries included segmental resection of the small bowel, right or left colectomy, total colectomy, sigmoidectomy, and proctocolectomy. Perianal surgeries included abscess drainage, seton insertion, and fistulotomy. Urgency procedures were considered for any case requiring surgery in less than 24 h. Examination under anesthesia without abscess drainage or seton insertion was excluded. Surgical recurrence included any surgical intervention for CD after the initial surgery. There were no restrictions on the type of surgery, location, phenotype, or time to recurrence. Both perianal and major abdominal surgeries after the first surgery were considered recurrence procedures. Planned/staged procedures such as staged pouches or anastomosis that were identified as second-stage procedures in the patient database were not considered surgical recurrences. During the initial diagnosis, the disease phenotype was described according to the Montreal classification. The four primary phenotype characteristics were age at diagnosis (A), topographic location (L), clinical behavior (B), and perineal location (P). This study was approved by the Teaching and Research Foundation of Distrito Federal (approval 2.475.561).

### Statistical analysis

The results were described by descriptive statistics and reported as averages and standard deviations (SDs). Nonparametric statistics were used throughout. Comparisons between groups were performed using Mann-Whitney U tests. Categorical variables were compared with χ^2^ tests. Cumulative incidence curves were calculated for each cohort by the Kaplan-Meier method and compared by log-rank tests. A multiple linear regression model was used for the main variables (ANOVA). The level of statistical significance was 5% (p<0.05). The statistical analysis was performed using Medcalc and SPSS. This study was approved by the Teaching and Research Foundation of Distrito Federal (number 2.475.561).

## RESULTS

### Study sample

The study cohort included 107 CD patients who had been evaluated from 2015 to 2021. Epidemiological data of surgical and clinical patients are shown in Table 1. The patient distribution by the interval between diagnosis and study entry (i.e., initial diagnosis) was 35.6% between 0 and 5 years, 23.7% between 6 and 10 years, 16.8% between 11 and 15 years, 16.8% between 16 and 20 years, 3.9% between 21 and 25 years, and 2.9% between 26 and 30 years. The mean age was 40.8±14 years. The mean duration of illness was 9.54±8.4 years. There is no statistical difference in gender between the clinical and surgery groups. At the time of data collection, 54% of patients used anti-TNF drugs; 35% used adalimumab, and 19% used infliximab. Extraintestinal manifestations had been diagnosed in 57.4%. The total mortality rate in the sample was 2% related to the surgical group.

**Table 1.** Clinical and demographic characteristics of the Crohn’s disease patients included in the study population.

Characteristics	Total n=107 (%)	Surgery n=58 (%)	Clinical n=49 (%)	P-value
Age (years, mean, and SD)	40.8±14	44.4±14	36.6±12	p=0.0028^#^
Female % (n=**69**)	64.4	38 (65.5)	31 (63.3)	p=0.813*
Male % (n=**38**)	35.6	20 (34.5)	18 (36.7)	p=0.813*
Illness duration, years, mean (SD)	9.54±8.4	12.6±7.4	6.48±4.7	p<0.0001^#^
Age at diagnosis, years	30.8±11	31.5±13	30.2±12	p=0.59^#^
Montreal phenotype classification				
L1 (Terminal ileum)	21 (19.6)	15(25.9)	6 (12.2)	p=0.076*
L2 (Colon)	34 (31.7)	16(27.6)	18 (36.7)	p=0.31*
L3 (Ileocolonic)	49 (45.7)	26(44.8)	23 (46.9)	p=0.82*
L4 (Upper GI)	3 (2.8)	1(1.7)	2 (4.1)	p=0.45*
Perianal disease	42 (39.2)	22 (37.9)	20 (40.8)	p=0.76*
Extraintestinal manifestations %	57.4	54.4	62.2	p=0.3*

^#^Mann-Whitney test; *χ^2^ test.

### Surgical incidence and epidemiological profile

Out of 107 patients, 58 (54.2%) underwent surgical procedures. The mean age of those who required surgery was 44.4±14 years compared with 36.7±12 years in those who received only clinical treatment (p=0.002). The highest percentage of patients submitted to surgery (22.8%) was over 40 years of age (p=0.01), and most elective surgical indications appeared over 10 years after diagnosis (60.7%). A multiple linear regression model was used to verify the association of the main variables. The model revealed a statistically significant analysis for age at diagnosis (R=0.276; t=2.94; p=0.004) (Table 2).

**Table 2.** Multiple linear regression for surgical treatment probability.

Multiple linear regression model										
Model	R	R^2^	R^2^ fitted	Standard error	Change statistics					Durbin-Watson
					R^2^ change	F Change	df1	df2	Sig. Change F	
1	0.276^a^	0.076	0.067	0.48342	0.076	8.654	1	105	0.004	
2	0.283^b^	0.080	0.062	0.48475	0.004	0.428	1	104	0.514	
3	0.297^c^	0.088	0.062	0.48485	0.008	0.957	1	103	0.330	1.879

Variables: age at diagnosis, perianal disease, and diagnosis time. Model 1: dependent variable: treatment (d); constant variable: age (a). Model 2: dependent variable: treatment (d); constants variables: age and perianal disease (b). Model 3: dependent variable: treatment (d); constants variables: age, perianal disease and diagnosis time (c).

### Crohn’s disease and surgical phenotypes

**Figure 1.**
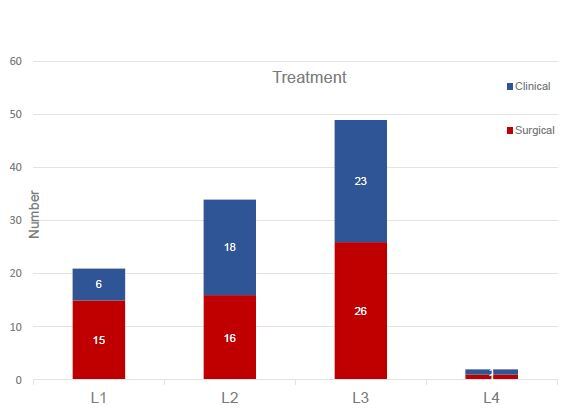
Medical (N=49) and surgical (N=58) treatment in Crohn’s disease patients with different Montreal L classification. The major risk was in L1 phenotype (RR=1.42; 95%CI 1.01–2.01; p=0.04).

The most frequent indication for abdominal surgery was L1 stricture disease with RR=1.42 (95%CI 1.0–2.0 (p=0.04)) (Figure 1). L2 Montreal phenotype had been associated with perineal disease, RR=1.49 (95%CI 1.03–2.16 (p=0.03)). L3 (31%) and L1 (20.6%) Montreal phenotypes were the main phenotypes with emergency surgical indications (n=30/58).

### Indications for surgery and types of surgical procedures

**Figure 2.**
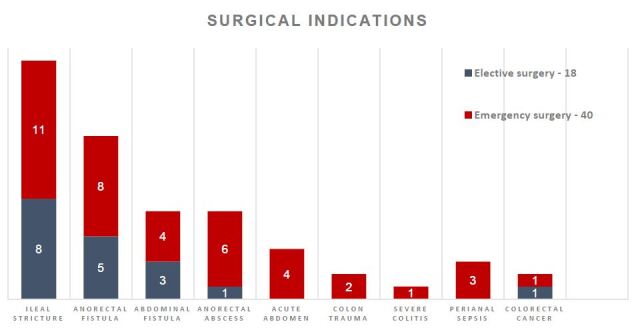
Surgical indications in 58 Crohn’s disease patients. Urgency surgery=40. Elective surgery=18.

**Figure 3.**
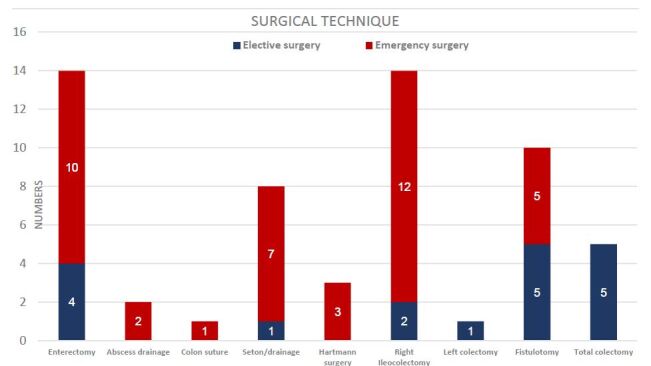
Surgical techniques performed in 58 Crohn’s disease patients.

The most frequent surgical indication was ileal stenosis in both L1 and L3 Montreal phenotypes, which accounted for 19 of the 58 procedures (32.7%). Eleven of the 19 were emergency procedures. Perineal abscess/sepsis (17.2%) and anorectal fistula (22.4%) were the most frequent indications of perianal disease. In these cases, 29.3% (17/58) were emergency procedures (Figure 2). The overall ostomy rate was 26.7%. Fecal diversion for perianal disease was the most common indication in 8.9% of the cases, and definitive ostomy was done in 3.1%. Enterectomy (24.1%) and right ileocolectomy (24.1%) were the most performed procedures in abdominal surgeries; however, right ileocolectomy was also the most performed procedure in emergency cases overall (Figure 3).

### Perianal surgery

The perianal disease had a prevalence coefficient of 39.3%. The average time of evolution to surgical treatment was 5.7 years after diagnosis of perianal disease, being the initial manifestation in 21.5%. One-third of the total surgical procedures were for perianal disease (Figure 3). The risk of emergency surgery in patients with perianal disease was increased for anorectal abscess and perianal sepsis (RR=1.43; 95%CI 1.21–1.7). The primary indication for anorectal fistula was not different in elective or emergency surgery (p=0.21). The mean age at emergency surgery was 41.28±14 years (p=0.4), 26 of the patients were men, and 25 were women. Surgical perianal disease was more likely to occur in L2 (47.6%) Montreal phenotype than the other phenotypes.

### Time until surgical procedure and surgery free time

The distribution of surgeries by time showed a bimodal curve. The initial peak (67.8%) occurred in patients with the main initial presentation of the disease as urgency, representing 11 cases of ileal stenosis, 8 cases of anorectal fistula, 6 cases of anorectal abscess, and 4 cases of acute abdomen (Figure 2).

**Figure 4.**
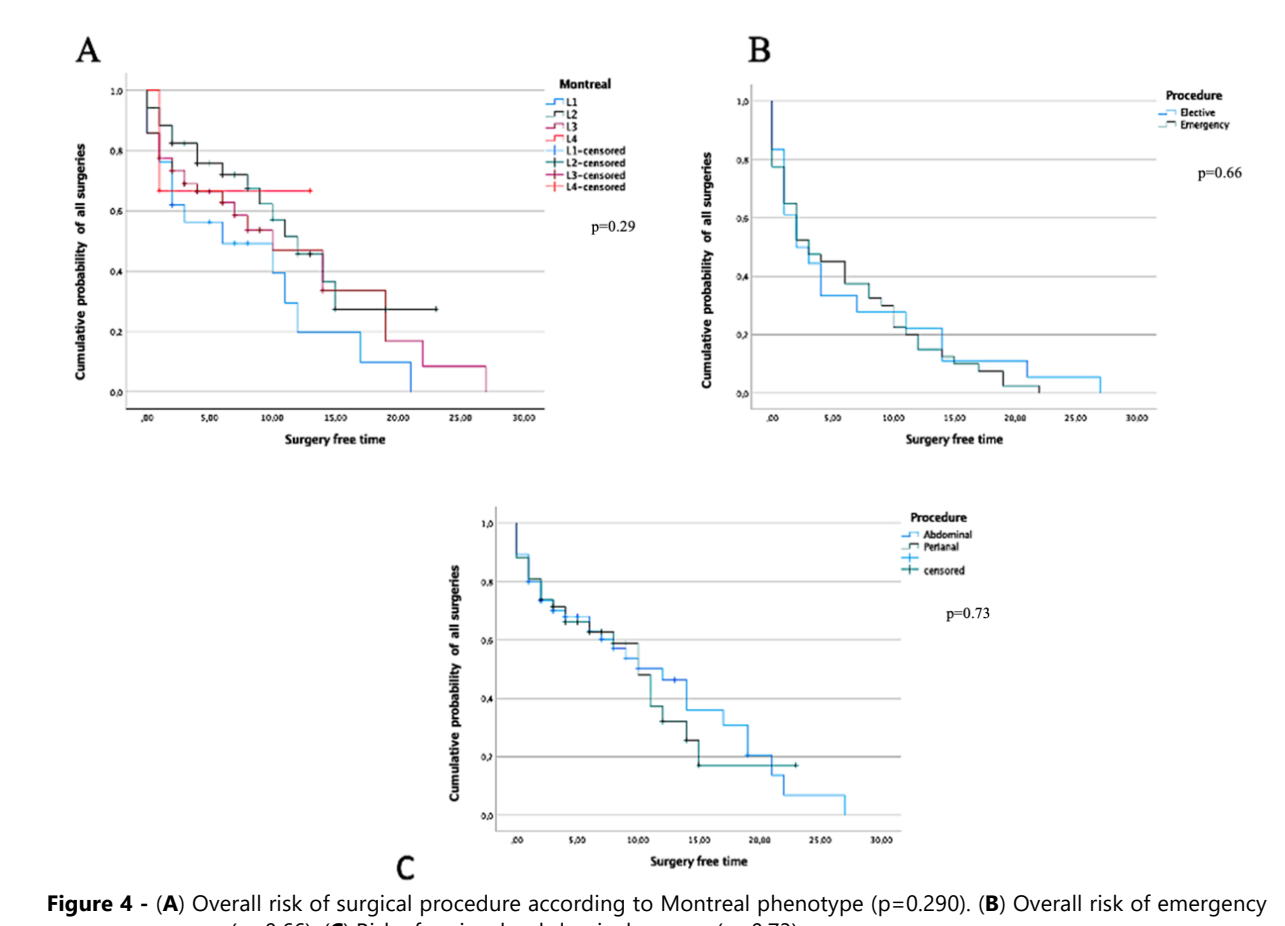
(**A**) Overall risk of surgical procedure according to Montreal phenotype (p=0.290). (**B**) Overall risk of emergency surgery (p=0.66). (**C**) Risk of perianal x abdominal surgery (p=0.73).

The second peak of surgical indications (32.1%) was seen over the first 11-year interval after diagnosis. Approximately half of those patients required emergency surgery, and the main indications were perianal disease with abscesses and fistulas. The surgery free time was studied with the Kaplan-Meier curve, and no difference was found between emergency, Montreal, and perianal variables through the log-rank (Mantel-Cox) test (Figure 4).

### Recurrence surgery and total procedures

A total of 96 surgical procedures were performed in 58 of the 107 patients. The overall risk of surgical recurrence was 51.7%. The main variable linked to recurrence was emergency indication (OD=2.1; 95%CI 1.6–6.6). Of these patients, most surgical recurrences were for perianal disease (46.5%). The mean time to surgery recurrence in this group was 1.8±2 years.

## DISCUSSION

The epidemiology of inflammatory bowel diseases in South America may uncover etiopathogenic factors due to its population diversity and geoecological conditions^16^. The recent rise of intestinal bowel disease (IBD) is well documented in developing countries; however, the explanation for this phenomenon is still under analysis^13^. This Brazilian retrospective specialist-based cohort study of 107 CD patients found that 96 surgical interventions had been performed in 58 patients.

Despite advances in the clinical management of CD, the rate of surgical intervention remains high around the world^11^. The overall risk of surgery varies by geographical region. The European population-based cohort studies from Norway, Hungary, and Denmark reported probabilities of surgical resection after diagnosis as 15, 30, and 38–52%, respectively^5,26,30^. In South America, Asia, and Africa, few reports in cohort studies showed the rate of surgery in CD.

In this Brazilian study, 54.2% of the patients with CD were treated surgically, with emergency surgery (68.9%) being the main indication in CD, which is higher than that reported in other studies^10,17^. The main common risk factor in this sample was the high prevalence of fibrostenotic disease particularly in the ileal/jejunal region, discovered mainly after an initial presentation of obstruction in the emergency room. Epidemiological factor analysis showed that the mean age at the time of surgery was significantly higher than that of the clinically managed patients (Table 1). In the multiple linear regression model, age at diagnosis was the predictor for surgery treatment (Table 2). Other studies reported that male gender was a predictor of surgery^4^, but that was not observed in this study.

Risk stratification to identify CD patients with high-clinical characteristics has been described. The Montreal classification has been used in studies worldwide to evaluate the potential predictors of surgical intervention and clinical response^2^. In this study, patients with the Montreal L1 fibrostenotic phenotype were more likely to be treated by surgery than other phenotypes (RR=1.42), being together L3 ileal fibrostenotic disease the main indication for emergency surgical treatment (Figure 1). Consequently, the main indications for abdominal surgery were ileal stricture (18.9%) and obstructive acute abdomen (6.89%) (Figure 2). Surgery was performed in 32.7% of the patients with ileal stricture, but only 12% of patients were operated for penetrating behavior. These results are in agreement with many other research cohorts worldwide^1,7,26,28^. Apparently, mesenteric involvement and fibrostenotic disease were common in L1 and L3 ileal diseases, and the initial intensity of the inflammatory process in the first episode together with the silent nature of this strictures seems to be decisive for the presentation of obstructive process in the emergency department.

Urgency surgical management due to obstruction or acute abdomen was small-bowel resection (17.2%) and right ileocolectomy (20.6%). Elective surgery for small-bowel obstruction including intestinal resection with primary anastomosis was the most common approach in this study, and resection with side-to-side anastomosis was used more often than end-to-end anastomosis. Although stricturoplasties are considered safe for the management of small-bowel stenosis, in general terms, resection of small-bowel disease was preferred in CD with active inflammatory phenotype, creeping fat presentation, or large segments affected, being the main reason for this approach in both emergency and elective procedures (Figure 3).

The possibility for an ostomy is a major concern for patients with CD, particularly those who may be at increased risk of a permanent ostomy^3^. Some studies found that nearly two-thirds of patients treated with fecal diversion remained diverted or required colectomy within 2 years^25,27^. The overall ostomy rate was 26.7% in our study. The main indications were fecal diversion for perianal disease (8.9%) and abdominal acute behavior (7.1%). Although fecal diversion was performed in emergency surgery to gain time, attempted reconstruction of bowel continuity was not possible at the time of data collection. Persistent inflammatory rectal disease and complex fistula with perineal sepsis were associated with failure to restore bowel continuity. Definitive ostomy was thus done in 3.1% of the procedures.

An important finding was the positive correlation between L2 and perianal disease leading to the consideration of those phenotypes as risk factors (RR=1.49). In this study, perianal disease was defined by perianal fistula, anorectal abscess, perianal sepsis, and stenosis disease. In this cohort, 39.6% of CD patients developed surgical perianal disease. The most common indication for emergency surgery in the entire cohort was anorectal abscess/perineal sepsis (15.5%). Patients with perianal CD were at increased risk of undergoing emergency surgery compared with non-perianal CD patients (RR=1.43). Some evidence indicates that combining surgery with medical therapy may benefit perianal fistula healing compared with surgery or clinical therapy alone^31^. However, if this complication is the initial manifestation of CD, it is predictive of surgery. Unfortunately, 8.6% of our patients presented with this as the initial form of the disease. In this cohort, there was no gender difference in this subgroup, but in some other studies, fistulizing perianal disease was more common in men^3,20^.

Recurrence surgery was the most significant complication with 51.7% overall risk. The main variable linked in these cases was emergency indication (OD 2.1). Of these patients, the most surgical procedures were done in perianal disease (46.5%). Most studies on perianal CD have focused on perianal fistulas. Recently, a Danish Nationwide cohort found only 9.7% of patients with perianal fistulas in CD. Another contemporary Dutch population-based study observed 13.9% of this disease^31^. Our tertiary hospital found a 15% of prevalence coefficient of perianal fistulas.

An important point in this study is the high initial manifestation of CD as an emergency. This fact reveals a consequence of an increased distribution of surgeries at the time of initial point. Hence, no difference was found between the cumulative probability of surgery free time for Montreal phenotypes, perianal disease, and emergency procedure (Figure 4).

There were several study limitations. It was not a population-based study but a hospital specialist-referred cohort, which led to the inclusion of patients with more severe and symptomatic diseases. The rate of emergency surgery (35.6%) was higher than other cohort studies, which may have influenced the overall rate of surgical intervention. Consequently, the overall risk of surgery must be interpreted with caution. Not all CD patients in this study had been evaluated by upper gastrointestinal endoscopy or magnetic resonance enterography making it impossible to confirm some behaviors or locations of disease. The follow-up of patients was relatively short, with only 5 years of observation.

## CONCLUSIONS

This cohort study showed that 54.2% of CD patients had been operated after diagnosis. The most frequent indication was ileal stenosis in both L1 and L3 Montreal phenotypes. Right ileocolectomy and enterectomy were the most performed abdominal surgeries. Perianal disease had a prevalence of 39.3% and one-third of the total surgical procedures. The main predictive risks for all procedure surgeries were age at diagnosis and fibrostenotic disease. The risk of emergency surgery in patients with perianal disease was increased as an initial presentation with anorectal abscess/perianal sepsis.
